# Genetic diversity of virus auxiliary metabolism genes associated with phosphorus metabolism in Napahai plateau wetland

**DOI:** 10.1038/s41598-023-28488-1

**Published:** 2023-02-24

**Authors:** Hang Yu, Lingling Xiong, Yanmei Li, Yunlin Wei, Qi Zhang, Haiyan Li, Wei Chen, Xiuling Ji

**Affiliations:** 1 Faculty of Life Science and Technology, Kunming University of Science and Technology, Kunming, China; 2 Medical School, Kunming University of Science and Technology, Kunming, China

**Keywords:** Ecology, Biogeochemistry

## Abstract

Viruses play important roles in ecosystems by interfering with the central metabolic pathways of the host during infection via the expression of auxiliary metabolic genes (AMGs), altering the productivity of ecosystems and thus affecting geochemical cycling. In this study, the genetic diversity of phosphorus metabolism AMGs *phoH*, *phoU* and *pstS* was investigated by phylogenetic analysis, PCoA analysis, and alpha diversity analysis based on metagenomic data. It was found that the majority of the sequences were unique to Napahai plateau wetland. It was shown that the genetic diversity of *phoH*, *phoU* and *pstS* genes was independent of both habitats and host origins. In addition, the metabolic pathway of AMGs associated with the phosphorus cycling was identified based on metagenomic data. When phosphorus is deficient, virus utilizes AMGs to affect the metabolic pathway, contributing to higher phosphorus levels in the host and facilitating virus survival, replication, and propagation in the host cell.

## Introduction

As the “kidneys of the earth”, wetlands have unique ecological functions and are the most active place for the energy flow and material circulation on earth. Napahai plateau wetland is located in the Three Parallel River Area of the Jinsha, Lancang and Nu rivers with unique geographic position, and possesses both terrestrial and aquatic characteristics. It belongs to a low-latitude (27° 47′ –27° 55′ ), high-altitude (average 3568 m), karst-landform seasonal swamp wetland, consisting of meadows, marshes, waters and forests. In addition, influenced by the southwest monsoon, Napahai plateau wetland forms a western monsoon climate in the low-temperate highland monsoon climate zone, with distinct dry and wet seasons. The unique geographic location, abundant water resources and rich nutrient lead to the rich biodiversity. However, due to geographical location, the species of Napahai plateau wetland are obviously different from other habitats, which determines the isolation, fragility and instability. Once affected by anthropogenic factors or external pressures, its ecosystem may undergo irreversible damage. The diversity of virus auxiliary metabolic genes (AMGs) can clarify the evolutionary history of organisms and the role they play in the ecosystem. However, the study on the genetic diversity of viral AMGs in plateau wetlands and how they regulate host metabolism has not been reported yet.

AMGs refers to a set of metabolism-related homologous genes carried by the viral genome^1,2^. AMGs include a number of genes that regulate host metabolism and life processes, and are currently divided into two main categories. The first class of AMGs is usually found in KEGG pathways (e.g. phosphate metabolism, carbon metabolism, nitrogen metabolism, sulfur metabolism, DNA biosynthesis, photosynthesis and other essential metabolic pathways^3,4^), i.e. AMGs that encode proteins with central metabolic functions. For example, genes involved in the electron transfer (*psaA*, *petE*, *petF*, *ptoX*, *speD*, *hli*) and light-harvesting processes (*ho1*, *pcyA*, *pebS*, *cpeT*), infected phage AMGs carrying photosynthetic system genes complements photosynthetic electron transport while transferring energy from the carbon fixation to the pentose phosphate pathway, leading to the increase of viral lysis^5,6^. Tsiola et al. conducted a metavirome study on coastal surface waters in the Ionian and South Aegean Seas, and revealed that viral AMGs participated in non-traditional energy production pathways (3HP, sulfur oxidation), Calvin cycle and TCA cycle^7^. Another class of AMGs does not usually appear in the KEGG pathway and encoded only proteins with general, unspecified metabolic roles or peripheral proteins involved in assembly and membrane transport functions. For example, AMGs associated with fatty acid metabolism in the *Myxoviridae* family can regulate host cytoplasmic or vesicle-like membrane fluidity. There are also AMGs involved in iron-sulfur cluster assembly, vitamin and cofactor synthesis, stress response, bacterial motility and chemotaxis, antioxidant and post-translational modifications^8^. In addition, AMGs are related to the community sensing, pathogenicity, host's defense, and spore formation involved in other ecosystems^9^. For example, abundant genes related to fatty acid metabolism and signaling were found in the viral genome, as well as those of fatty acid metabolism subsystem and components of the 3-hydroxypropionic acid (3HP) cycle. Phages that infect *Prochlorococcus* carried the phosphate acquisition gene *pstS*, which promoted phosphorus uptake by the host during infection^10^. Sabri et al. identified the gene *que* in the phage genome which could redirect host protein synthesis, enhanced the translation efficiency of the host, resulting in more efficient assembly of progeny phages^11^. The high abundance and genetic diversity of viral AMGs in the ocean suggested that virus played a significant role in microbial ecology by influencing the host's cellular functions via regulating host metabolism.

Phosphorus (P), an essential macronutrient for all life on Earth, has long been considered the second most limiting nutrient in some terrestrial ecosystems after nitrogen (N)^12^. In addition to being essential for DNA replication and nucleotide biosynthesis, it also critical for microbial growth. Unfortunately, there is growing evidence that P limitation is a general phenomenon occurring in various types of terrestrial ecosystems around the world, rather than in some specific terrestrial ecosystems^13,14^. The major viral AMGs currently associate with phosphorus metabolism include *phoH*, *phoA*, *phoU*, *phoB*, *phoR*, *pstS*, *phoE*, and *ugpQ* etc. Some phosphorus acquisition genes such as *pstS* and *phoH*, and alkaline phosphatase synthesis gene *phoA* existe in phages genome, which are beneficial to host cells to enhance phosphorus uptake and transport under low phosphorus environment. The *phoH* gene belongs to Pho regulon and is widely distributed in prokaryotes and viruses^15^. Li et al. identified *phoH* genes associated with phosphate metabolism in Northeastern wetland, which could be used as an effective biomarker gene^16^. To date, reports on phosphate metabolism-associated viral AMGs have focused on marine, lake, and rice paddy habitats. Few studies have been performed on phosphorus metabolism-associated viral AMGs in wetlands, especially plateau wetlands. In this paper, three genes related to phosphorus metabolism, *phoH*, *phoU* and *pstS* were obtained based on metagenomic data, which are expressed only in low or relatively phosphorus-deficient environments and thus regulate phosphorus homeostasis. Compared with the phosphorus content of other wetlands (Table S1 in the Supplementary Material), Napahai plateau wetland showed a low phosphorus level, so *phoH*, *phoU* and *pstS* were selected in this study.

In this paper, we investigated the genetic diversity of *phoH*, *phoU*, and *pstS* genes in Napahai plateau wetland based on metagenomic data by using phylogenetic analysis, PCoA analysis, and α-diversity analysis. We gained a brief understanding of the metabolic pathways AMGs involved in. It provides the foundation for subsequent studies on the genetic diversity of other metabolism-related AMGs in Napahai plateau wetland and the regulation of host metabolic pathways by viral AMGs, as well as virus-host co-evolution.

## Results and discussion

### Screening for viral AMGs

Viral protein annotation using VIBRANT and DRAM-v software combined with manual proofreading identified the viral AMGs in Napahai plateau wetland, including the viral AMGs *phoH*, *phoU* and *pstS*, which were associated with phosphorus metabolism.

### Phylogenetic analysis of AMGs associated with phosphorus metabolism in Napahai plateau wetland

**Figure 1:**
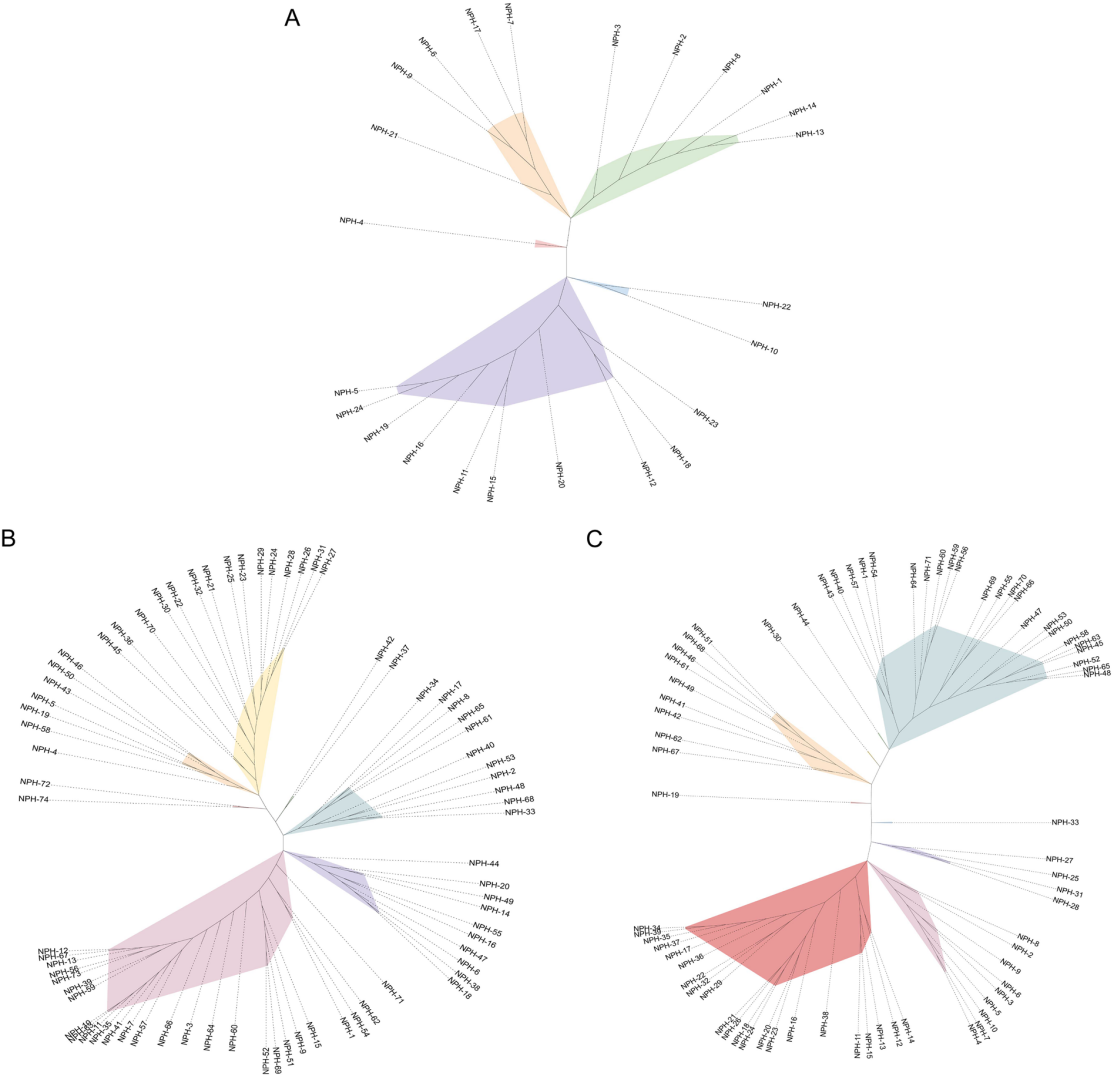
Phylogenetic analysis of phosphorus metabolism AMGs in Napahai plateau wetland, different colors represent different branches. (**A**) Phylogenetic analysis of *phoH* genes. (**B**) Phylogenetic analysis of *phoU* genes. (**C**) Phylogenetic analysis of *pstS* genes.

There were 24 amino acid sequences of *phoH* gene in Napahai plateau wetland (Fig. 1A). They were divided into 5 clusters, the largest of which had 10 sequences, while the smallest cluster had only 1 sequence. The remaining 3 clusters contained 6, 5 and 2 sequences, respectively. The *phoH* gene was genetically diverse in Napahai plateau wetland, which might be related to the different host origins. A total of 74 sequences of *phoU* gene could be found in seven clusters (Fig. 1B), with the largest cluster containing 27 sequences and the smallest cluster having two sequences. Similar to *phoH*, *phoU* was also genetically diverse, but richer than that of *phoH*. There were 71 *pstS* sequences forming 9 clusters, with the largest cluster of 23 sequences and the smallest cluster only 1 sequence (Fig. 1C). It could be seen that the genetic diversity of *pstS* was better than that of *phoH* and *phoU*, which might be related to the unique geographical location. Napahai plateau wetland is located in the Three Parallel Rivers of Yunnan protected areas, which forms a complex landscape, and then controls the evolution and characteristics of organisms, thus showing abundant biodiversity. Li et al. obtained 58 *phoH* gene sequences from Northeastern wetland sediments of China, which were 22%–99% consistent at the amino acid level, and found that the *phoH* gene could regulate phosphate uptake and metabolism under the low phosphate or phosphate limitation conditions^16^. However, the exact function remained unclear. The *phoH* gene clustered into five clusters in Napahai plateau wetland, indicating high genetic diversity. Additionally, water and soil samples were collected from eight separate sampling sites, and there were differences between samples environments, which might also have an impact on the genetic diversity of the three genes.

### Phylogenetic analysis and PCoA analysis of AMGs associated with phosphorus metabolism from different habitats and host origins

**Figure 2:**
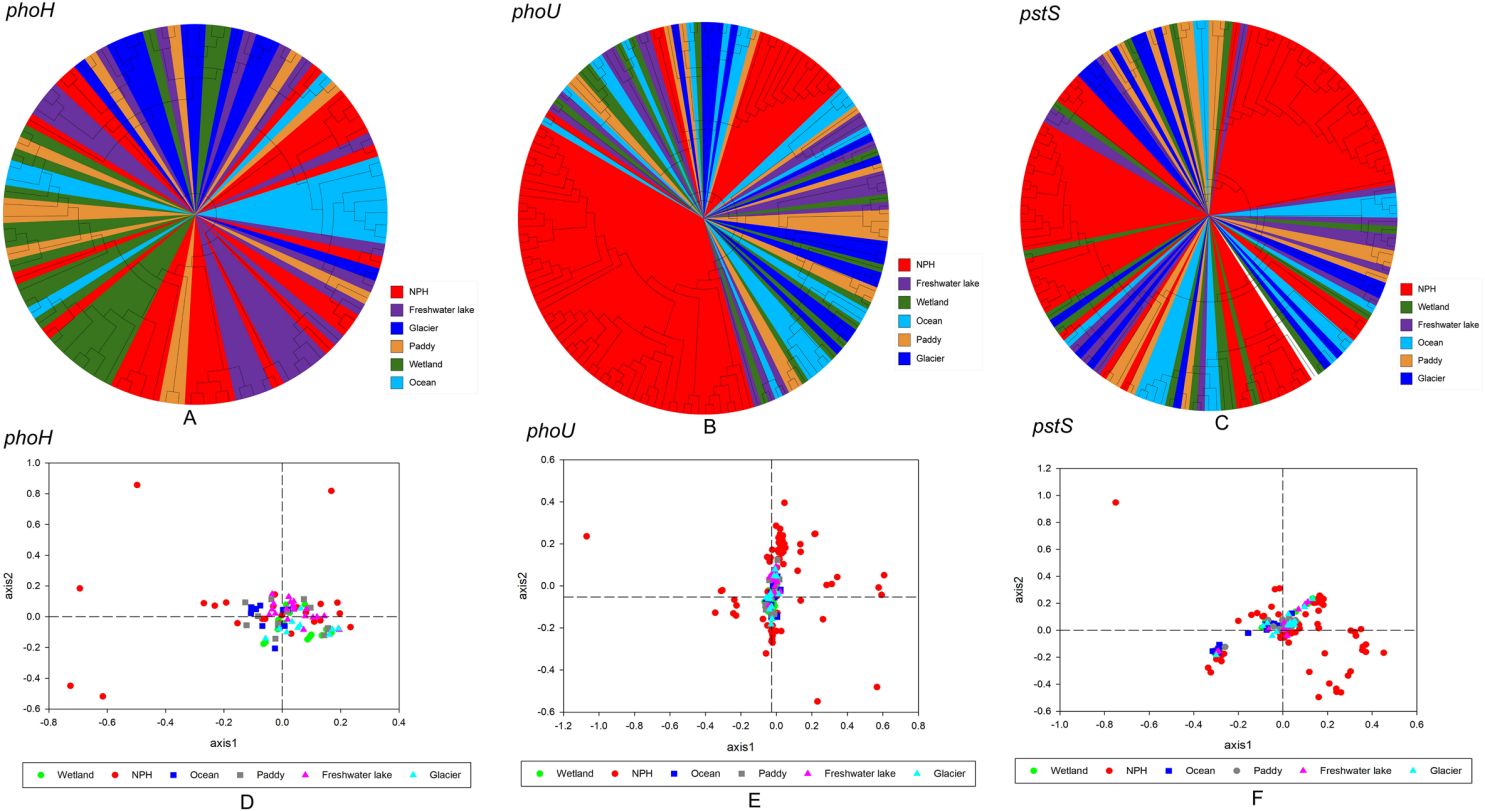
Phylogenetic analysis and PCoA of phosphorus metabolism AMGs in different habitats, different colors represent different habitats. (**A**) Phylogenetic analysis of *phoH* genes in different habitats. (**B**) Phylogenetic analysis of *phoU* genes in different habitats. (**C**) Phylogenetic analysis of *pstS* genes in different habitats. **(D**) PCoA analysis of *phoH* genes in different habitats. (**E**) PCoA analysis of *phoU* genes in different habitats. (**F**) PCoA analysis of *pstS* genes in different habitats.

In order to understand the genetic diversity of viral AMGs (*phoH*, *phoU*, *pstS*) associated with phosphorus metabolism in Napahai plateau wetland, a phylogenetic tree of phosphorus metabolism AMGs from different habitats was constructed, and PCoA analysis was performed (Fig. 2). The results showed that most sequences of *phoH*, *phoU* and *pstS* genes in Napahai plateau wetland clustered individually, especially *phoU* and *pstS* genes, and only a few sequences were closely related to those of other habitats. In Fig. 2A, 14 sequences clustered individually and were relatively far from sequences of other habitats, whereas 7 sequences were close to those from freshwater lakes, and other 3 sequences were close to those from rice fields, oceans and other wetlands, respectively. Therefore, the genetic diversity of *phoH* in Napahai plateau wetland was independent of the habitat. Moreover, some of the *phoH* sequences were clustered with those of other habitats and distributed in the fourth quadrants (Fig. 2D). From Fig. 2B, apart from 3 sequences which clustered with those from the marine habitats and freshwater lakes, the rest were clustered separately. Whereas in Fig. 2E, apart from only a few sequences, most sequences of *phoU* were far away from those of different habitats, which was consistent with Fig. 2B. Thus, the genetic diversity of *phoU* gene in Napahai wetland was also independent of habitat, where the separately clustered sequences may be unique. From Fig. 2C, we can seen that apart from 8 sequences which more closely related to those from the freshwater lake, ocean, rice field, and other wetlands, all the rest were individually clustered. The result was consistent with that of Fig. 2F. Therefore, the genetic diversity of the *pstS* gene was also habitat-independent.

**Figure 3:**
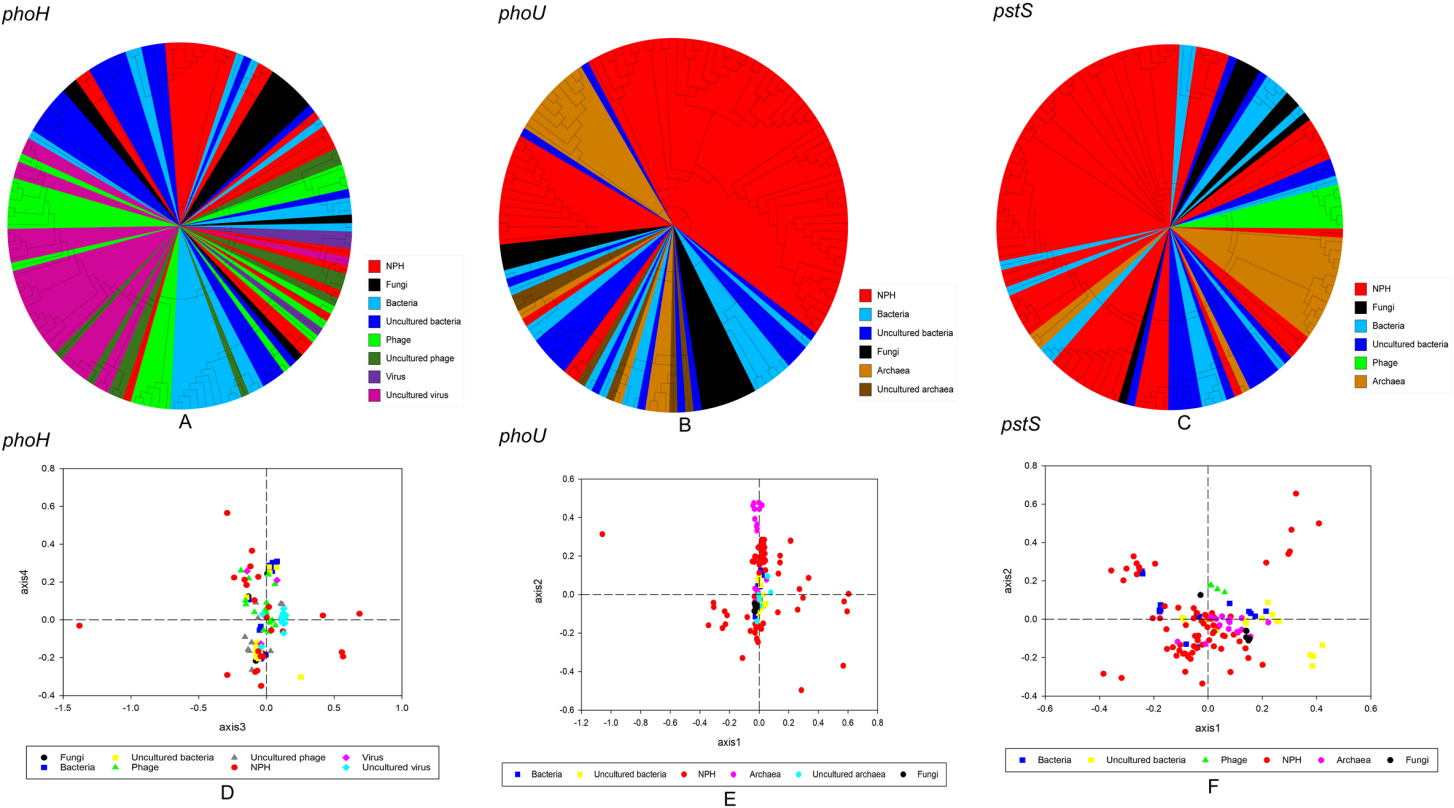
Phylogenetic analysis and PCoA of phosphorus metabolism AMGs from different host origins, different colors represent different host origins. (**A)** Phylogenetic analysis of *phoH* gene from different host origins. (**B**) Phylogenetic analysis of *phoU* gene from different host origins. (**C**) Phylogenetic analysis of *pstS* gene from different host origins. (**D**) PCoA analysis of *phoH* genes from different host origins. (**E**) PCoA analysis of *phoU* genes from different host origins. (**F**) PCoA analysis of *pstS* genes from different host origins.

To study whether the genetic diversity was related to host origins, three AMGs associated with phosphorus metabolism were selected for phylogenetic and PCoA analyses with AMGs sequences from different host origins (Fig. 3). It showed that some sequences of all three genes were similar to those from different host origins, while the remaining were separately clustered. In Fig. 3A, apart from 14 sequences which clustered with those from fungi, bacteria, non-culturable phages, phages and viruses, all the rest were clustered separately. Whereas, most sequences were clustered with those from different host origins together, and only six sequences were far from other sequences of different host origins based on PCoA analysis (Fig. 3D). Only three sequences were clustered with those of archaea and uncultured archaea, and the rest were clustered together to form independent clusters (Fig. 3B). A small amount of sequences were gathered with bacteria, uncultured bacteria, archaea and uncultured archaea, and the rest were clustered individually (Fig. 3E). As can be seen in Fig. 3C, six sequences were clustered with those of archaea, fungi, bacteria, while the rest were clustered separately. Some sequences were gathered with bacteria, uncultured bacteria, archaea and uncultured archaea, and others were clustered separately (Fig. 3F). PCoA analysis was largely consistent with phylogenetic analysis. So the genetic diversity of *phoH*, *phoU* and *pstS* genes in Napahai plateau wetland was independent of the host origins.

Overall, the genetic diversity of *phoH*, *phoU* and *pstS* genes associated with phosphorus metabolism in Napahai plateau wetland was independent of both the habitats and host origins based on phylogenetic and PCoA analyses. It suggested that three genes showed relatively rich genetic diversity and were not genetically limited by differences in habitats or host origins. Han et al. showed that *phoH* sequences were widely distributed in soil, freshwater, and seawater environments in different locations around the world, indicating the genetic diversity independent of the environment^17^, which corroborated the conclusions in our study. Phylogenetic analysis of the 58 viral *phoH* gene sequences in Northeastern wetland of China revealed that some sequences were clustered with bacterial sequences and others clustered with phages sequences^16^. In Napahai plateau wetland, some *phoH* gene sequences were clustered with fungal, bacterial, phage, uncultured phage, and viruses. Hence, the genetic diversity of *phoH* gene was independent of the host origins in either Northeastern wetland or Napahai plateau wetland. Compared with Northeastern wetland, the *phoH* genes in Napahai plateau wetland showed more abundant genetic diversity, which may be related to geographical location and climate. Additionally, compared with sequences from different habitats and host sources, partial sequences from Napahai plateau wetland were clustered individually, thus they were unique, which might be related to the unique geography. Napahai plateau wetland is located in the Three Parallel Rivers with low latitude and high altitude, and shows specific characteristics which not found in other habitats, and then the species very different, thus providing the possibility for the emergence of unique genetic sequences. Of course, it would require further verification by subsequent study.

As far as the current studies are concerned, most reports on phosphorus AMGs focused on the function. Wang et al. mentioned that the *phoH* gene regulated phosphate uptake or metabolism under the low phosphorus or phosphate limitation conditions^18^. Kelly et al. isolated several phages from oligotrophic water bodies with low phosphorus condition, found that they contained the phosphate binding transporter gene *pstS* by sequencing, which enhanced the host cell with increasing the infection cycle of phages by increasing phosphate utilization^19^. Gardner et al. studied the *PhoR-PhoB* two-component regulatory system in *E. coli*, which regulated the expression of relevant genes according to environmental phosphate concentration and enabled cells to adapt the phosphate starvation^20^. The *phoU* existed in many bacteria and was identified as an auxiliary protein of the phosphate-specific transporter system, regulating phosphate metabolism in the host cell acting as phosphate regulators^21^. Few studies had been conducted on its genetic diversity, therefore, the information on the genetic diversity was relatively scarce.

### α diversity analysis of phosphorus metabolism AMGs in different habitats and different host origins

Chao, Shannon and Simpson diversity indices are common mathematical measure of species alpha diversity in the community. Chao focuses on species richness. Shannon index and Simpson index measure species richness and evenness. Simpson reinforces evenness and Shannon reinforces richness^22^.

**Figure 4:**
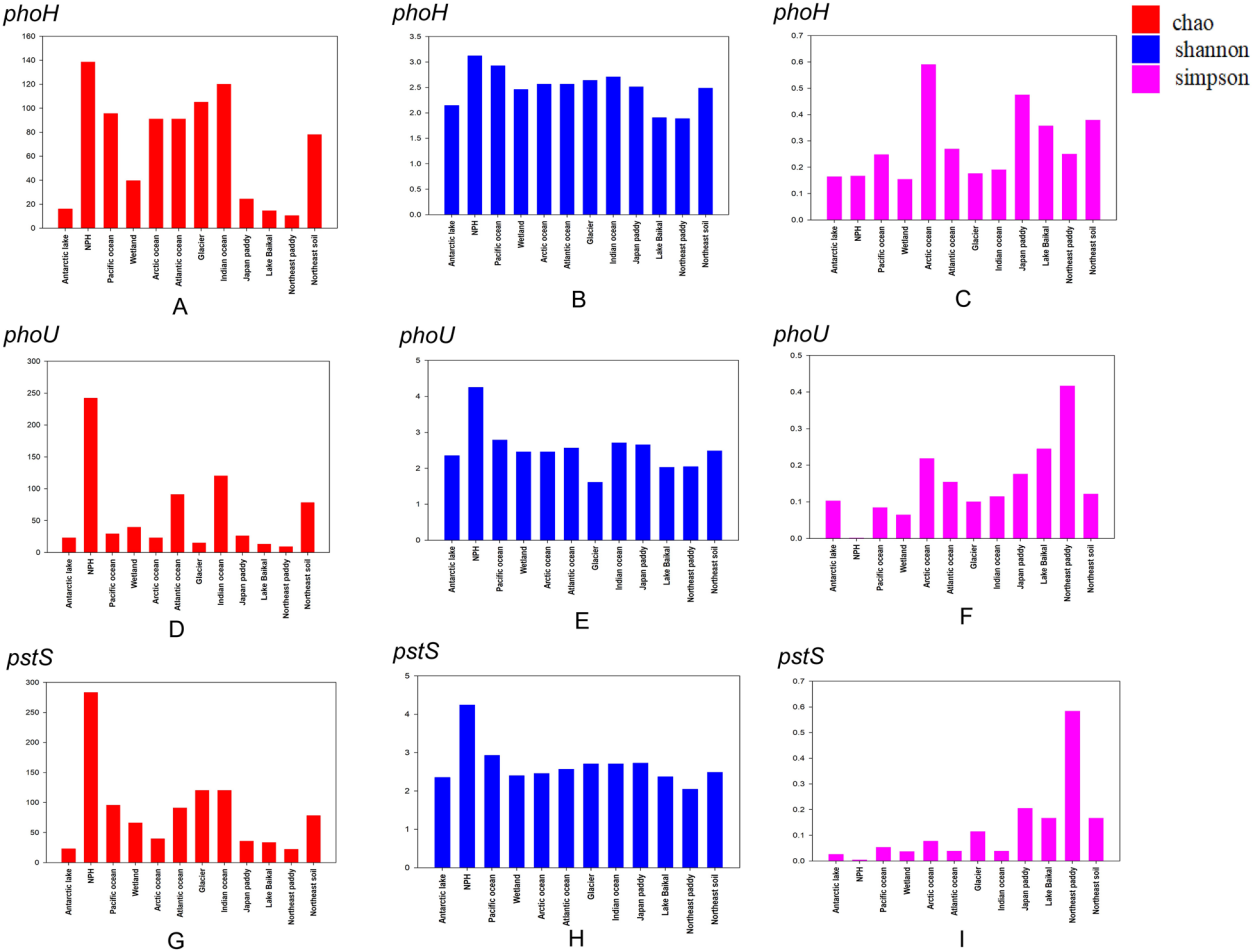
Plots of genetic diversity indices analysis of phosphorus metabolism AMGs in different habitats, different colors represent different genetic diversity indices. (**A**, **D**, **G**) Represent respectively the Chao values of *phoH*, *phoU*, and *pstS* genes in different habitats. (**B**, **E**, **H**) Represent respectively the Shannon values of *phoH*, *phoU*, and *pstS* genes in different habitats. (**C**, **F**, **I**) Represent respectively the Simpson values of *phoH*, *phoU*, and *pstS* genes in different habitats.

Sequences from different habitats, such as Napahai plateau wetland, Pacific Ocean, Lake Baikal, Northeast rice fields, glaciers, and wetlands, were selected for α-diversity analysis (Fig. 4). The genetic diversity indices, such as Chao, Shannon and Simpson, calculated based on the OUT dataset, were used to characterize the alpha diversity. Among them, larger Chao values, smaller Simpson values or larger Shannon values indicate higher genetic diversity. Only at the level of Chao values (Fig. 4A,D,G) and Shannon values (Fig. 4B,E,H), the values of *phoH*, *phoU*, and *pstS* in Napahai plateau wetland were greater than those from other habitats, indicating better heritable, which might be related to the unique geographical location and abundant water resources. The geographical location made it unique and less influenced by external factors, and abundant water resources created a rich biodiversity, thus providing a good genetic environment. From the Simpson values (Fig. 4C,F,I), the values of *phoU* and *pstS* genes were smaller than those of other habitats, indicating better inherited. For the *phoH* gene, the Simpson value was closer in magnitude and lower than those in Antarctic Lake and wetlands, indicating better heritable.

**Figure 5:**
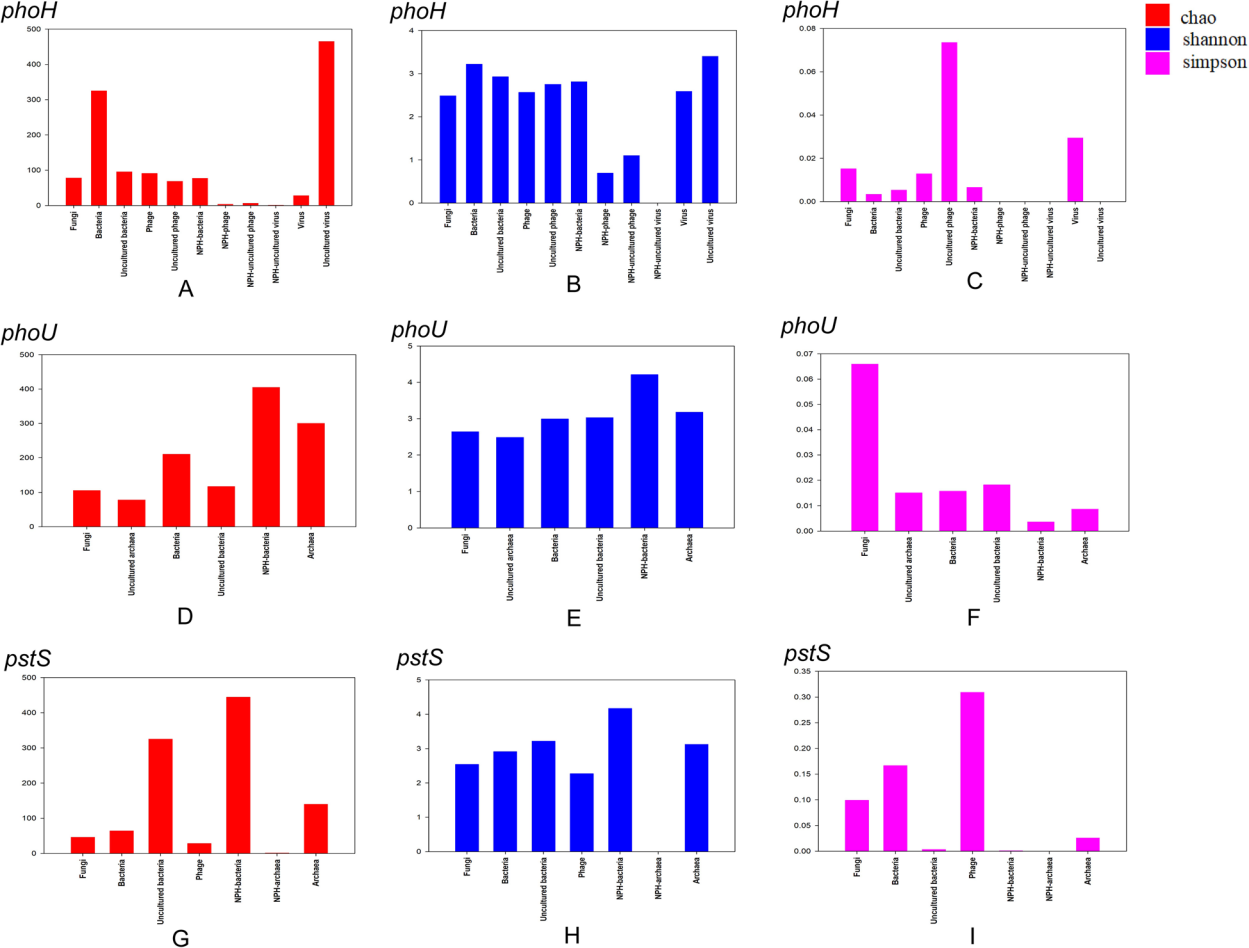
Plots of genetic diversity indices analysis of phosphorus metabolism AMGs from different host origins, different colors represent different genetic diversity indices. (**A, D, G**) Represent respectively the Chao values of *phoH*, *phoU*, and *pstS* genes from different host origins. (**B**, **E**, **H**) Represent respectively the Shannon values of *phoH*, *phoU*, and *pstS* genes from different host origins. (**C**, **F**, **I**) Represent respectively the Simpson values of *phoH*, *phoU*, and *pstS* genes from different host origins.

Three AMGs associated with phosphorus metabolism in Napahai plateau wetland were selected for α-diversity analysis with AMGs sequences from different host origins (Fig. 5). In Fig. 5A, the Chao values of *phoH* gene from bacteria, phages, uncultured phages and uncultured viruses in Napahai plateau wetland were smaller than those of bacteria, phages, uncultured phages and uncultured viruses, indicating the poor genetic diversity. In addition, compared to the genetic diversity of sequences from other host sources, the genetic diversity of *phoH* gene from bacteria in Napahai plateau wetland was better. As can be seen in Fig. 5D, G, the Chao values of *phoU* and *pstS* genes from bacteria in Napahai plateau wetland were greater than those of other host origins, indicating better genetic diversity, while the Chao values of *pstS* genes from archaea in Napahai plateau wetland were smaller than those of other host origins, indicating poor genetic diversity.

The Shannon value of *phoH* gene from bacteria in Napahai plateau wetland was smaller than that of bacteria and uncultured viruses, indicating poor diversity, but larger than other host sources, indicating better genetic diversity (Fig. 5B). The Shannon values of *phoH* gene from phages and uncultured phages in Napahai plateau wetland were lower than those of other host origins, indicating poor diversity. The Shannon value of *phoH* genes from uncultured viruses in Napahai plateau wetland was 0, probably due to sample size too small to calculate the Shannon value. In Fig. 5E, H, the Chao values of *phoU* and *pstS* genes from bacteria in Napahai plateaus wetland were greater than those from other host sources, indicating better diversity, while the Shannon value of *pstS* gene from archaea in the Napahai plateau wetland was 0, probably small sample size.

The Simpson values of *phoH* genes from phage, uncultured phage and uncultured virus in Napahai plateau wetland were smaller than those of other host origins (except uncultured virus), indicating better diversity. The smaller Simpson values of *phoH* genes related to fungi, phages, uncultured phages, and viruses indicated better diversity, while the larger Simpson values compared to bacteria, phages, and uncultured viruses indicated poor diversity (Fig. 5C). As can be seen in Figs. 5F,I, the Simpson values of *phoU* genes from bacteria and *pstS* genes from bacteria and archaea in Napahai plateau wetland were smaller than those of other host origins, indicating better genetic diversity.

Currently, most studies on phosphorus AMGs employed phylogenetic analysis^16,23^. In contrast, relatively few AMGs associated with phosphorus had been reported based on α-diversity analysis, so it was difficult to obtain specific values of α-diversity indices in other studies.

### Biogeochemical cycling of AMGs associated with phosphorus metabolism in Napahai plateau wetland

**Figure 6:**
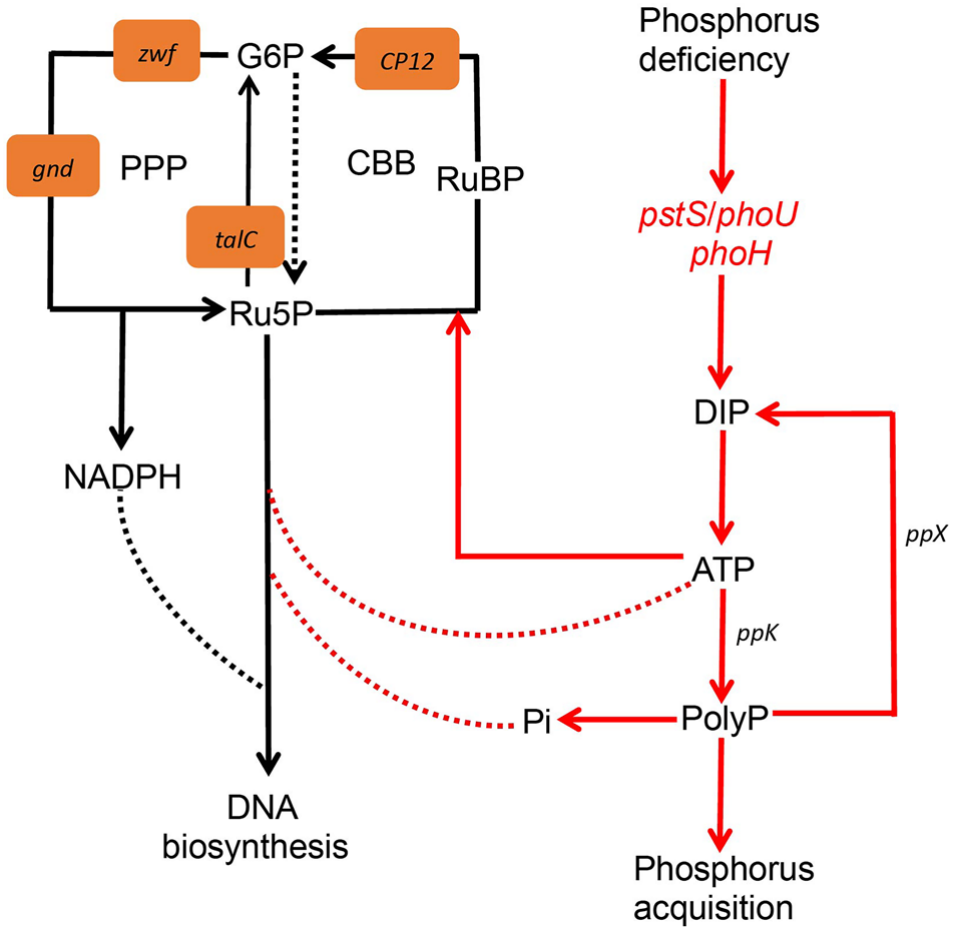
Biogeochemical cycling of AMGs associated with phosphorus metabolism in Napahai plateau wetland. Red line indicates the process of phosphorus metabolism.

Viruses are the gene carriers in susceptible hosts, and AMGs introduced by viruses into new hosts can enhance viral replication and/or influence key microbial metabolic pathways of the biogeochemical cycles^24^. It is well known that phosphorus is an essential nutrient and plays essential roles in cells^25^. Phosphorus deficiency leads to restricted cell division, down-regulation of photosynthesis, reduced protein and nitrogen content and chlorophyll synthesis^26^. To study the effect of AMGs associated with phosphorus metabolism, a phosphorus metabolic pathway containing *phoH*, *phoU* and *pstS* genes was constructed based on metagenomic data (Fig. 6). When phosphorus deficiency occurs in the host, it leads to the expression of *phoH*, *phoU* and *pstS* genes. *phoH* is a phosphate starvation inducible gene, while *pstS* acts as a phosphate transport gene and *phoU* belongs to a phosphate regulatory gene that produces dissolved inorganic phosphorus (DIPs), which then undergoes a series of reactions to produce ATP. The generated ATP becomes PolyP under the action of *ppK* which encoding polyphosphate kinase, or is used in Calvin cycle to provide energy for Ru5P to produce RuBP, or is used for DNA biosynthesis to provide energy. PolyP is regenerate into DIP with *ppX* which encoding exopolyphosphatase, and also involves in the biosynthesis process of DNA as Pi to provide phosphate for the nucleic acids synthesis. Thus, phosphorus metabolism of AMGs invoved plays a significant role in the life process of the virus and host. In addition, *phoE* and *ugpQ* genes also are identified in Napahai plateau wetland, but their roles in the phosphorus cycling are currently unknown and need further study.

Based on the phylogenetic and PCoA analyses, we found that the *phoH*, *phoU*, and *pstS* genes all showed unique sequences, which might be drive the microorganisms to produce the phosphorus metabolic pathway in Napahai plateau wetland. Of course, in order to prove this pathway, further validation might be done by metabolomics or metabolic flow method. Furthermore, the phosphorus metabolic pathway was poorly reported, so we could not compare with the phosphorus pathway from other environment to find commonalities and differences.

## Materials and methods

### Sample collection

According to the principle of typicality, water and soil samples were collected in Napahai plateau wetland, and eight sample points were chosen to sample soil. The depth of the soil layer at the sampling points was about 5–8 cm^27^, and 5 kg of soil samples were collected from each sample point. All the soil samples were mixed and divided into three portions, denoted as S1, S2 and S3. Water samples were collected along the lake, a total of eight sampling points. All water samples were mixed and divided equally into three parts as three replicates recorded as W1, W2 and W3. The latitude, longitude, altitude, air temperature, atmospheric pressure and pH value were recorded.

### Nucleic acid extraction and high-throughput sequencing

The DNA of soil and water samples was extracted by QIAamp DNA kit (Qiagen), and the quality of the separated and purified DNA was examined and evaluated using 0.8% agarose gel electrophoresis and Nano Drop spectrophotometer. When a single band appeared on the gel without trailing, and the concentration reached 50 ng/μL, it could be used for subsequent analysis.

The fragments were randomly broken by ultrasonic fragmentation, and the sticky ends were repaired to flush ends by T4 Polymerase, Klenow DNA polymerase and T4 PNK, and then the ligated products were recovered with the addition of special Adapter ligation. The electrophoresis method was selected to recover the target fragment of about 300 bp in size. The DNA fragments with Adapter at both ends were amplified using PCR technique^28–30^. The qualified library was prepared and sequenced, and 125 bp Paired-End double-end sequencing was performed using HiSeq 2500 high-throughput sequencing platform with an insertion length of 300 bp by Guangzhou Magigene Biotechnology Co., Ltd.

### Screening for viral AMGs

Firstly, CheckV^31^ was employed to distinguish and eliminate host contamination based on gene content for a single sequence. Then, based on VirSorter v2.0^32^, viral contigs were further DRAM-v annotation^33^, and the required input files of DRAM-v were engendered. Next, the chosen viral contigs were worked through DRAM-v using default parameters, with gene definitions and auxiliary scores of < 4 considered putative AMGs. Conserved domains of viral AMGs were detected using the NCBI CD-search tool^34^. The Phyre2 web portal was used to search the protein structural homology^35^. Moreover, VIBRANT^36^ annotations were made on viral contigs based on KEGG using default parameters. KEGG annotations categorized into “metabolic pathways” were reported as potential AMGs^36^. No curations were executed on the VIBRANT output.

### Phylogenetic analysis of AMGs associated with phosphorus metabolism in Napahai plateau wetland

The amino acid sequences of AMGs associated with phosphorus metabolism in Napahai plateau wetland were searched based on metagenomic data. The phylogenetic tree was constructed using the maximum likelihood method in MEGA X software^37^ with Bootstrap of 1000. To study the amino acid sequences of *phoH*, *phoU*, and *pstS* from Napahai plateau wetland with other habitats (including the ocean, lake, rice field, wetland, glacier, etc.) and other host origins (including bacteria, uncultured bacteria, fungi, phage, uncultured phages, viruses, uncultured viruses, archaea and uncultured archaea, etc.) as references, the amino acid sequences of *phoH*, *phoU* and *pstS* were sampled 1000 times at the amino acid level using the maximum likelihood method in MEGA X software to construct phylogenetic trees. Amino acid sequences information of *phoH*, *phoU*, and *pstS* from other habitats and host sources were detailed in Tables S2–S7 in the Supplementary Material.

### PCoA analysis of AMGs associated with phosphorus metabolism in Napahai plateau wetland

Principal co-ordinates analysis (PCoA) is a non-constrained method of data dimensionality reduction analysis that can be used to study the similarity or difference of samples^38,39^. The nucleotide sequences corresponding to the amino acid sequences of different origins and host sources searched from NCBI were selected, and other specific nucleotide sequence information of different habitats and host sources can be referred to Tables S8–S13 in the Supplementary Material. Combined with the nucleotide sequences of *phoH*, *phoU*, and *pstS* from Napahai plateau wetland, the UniFrac distances of all *phoH*, *phoU*, and *pstS* nucleotide sequences were calculated using Mothur software^34^, and then the PCoA was plotted using the mapping software Sigma Plot 11.0.

### α diversity analysis of AMGs associated with phosphorus metabolism in Napahai plateau wetland

The nucleotide sequences of *phoH*, *phoU* and *pstS* from different habitats and host sources were searched from NCBI, and specific information on nucleotide sequences could be found in Tables S8–S13 in the Supplementary Material. Combined with sequences from Napahai plateau wetland, the OTUs were typed using Mothur software, and genetic diversity indices, such as Chao, Shannon and Simpson indices, were used to characterize α-diversity.

### Biogeochemical cycling of AMGs associated with phosphorus metabolism in Napahai plateau wetland

Based on the metagenomic data, the enzymes and their functions of AMGs associated with phosphorus metabolism were searched by KEGG to analyze the phosphorus cycling in Napahai plateau wetland.

## Supplementary Information

Supplementary Information 1.

## Data Availability

The datasets generated during the current study are available from the corresponding author on reasonable request.
