# Supplementary material for: Genetic diversity of virus auxiliary metabolism genes associated with phosphorus metabolism in Napahai plateau wetland
Source: Sci Rep. 2023 Feb 24;13:3250. doi: 10.1038/s41598-023-28488-1 (PMC9958192; doi:10.1038/s41598-023-28488-1)
Supplement: Supplementary file 1 — Supplementary Information 1. [file 41598_2023_28488_MOESM1_ESM.docx]

**Table S1 Phosphorus content in different wetlands**

| Wetland name | Phosphorus content(mg/kg) |
| --- | --- |
| Napahai Plateau Wetland | 3.7 ～5.7 |
| Erhai Wetland | 3300 |
| Pearl River Estuary Wetland | 335.8 |
| Yinchuan Plain Wetland | 1080 |
| Eastern Jilin Mountain Swamp Wetland | 4000 |
| Liaohe estuary Wetland | 660 |
| Baotou Yellow River Wetland, Inner Mongolia | 980 |
| Jiaozhou Bay Reed Wetland | 150 |
| Jiaozhou Bay Alkaline Pine Wetland | 660 |

**Table S2 Nucleotide sequence fragment information of *phoH* gene in different habitats**

| Habitat | GenBank ID | Number of sequences |
| --- | --- | --- |
| Pacific ocean | CP003179 CP081862 CP002344 CP091912 | 20 |
|  | CP000127 CP047851 CP011011 CP011030 |  |
|  | CP002361 CP003257 CP002344 CP014782 |  |
|  | CP031165 CP004387 CP029789 CP038852 |  |
|  | CP090836 CP029151 CP014796 CP029488 |  |
| Atlantic ocean | CP051248 HQ337021 CP051251 CP041159 | 13 |
|  | CP019124 CP031585 CP021404 CP033580 |  |
|  | CP054599 CP104533 CP003946 CP002543 |  |
|  | CP040367 |  |
| Arctic ocean | CP010797 CP027434 CP090837 CP041036 | 13 |
|  | CP017965 CP071868 CP026490 CP072852 |  |
|  | CP000447 CP011494 CP003735 CP011025 |  |
|  | CP031848 |  |
| Indian ocean | CP091056 CP002683 CP038256 CP041614 | 15 |
|  | CP031598 CP042862 CP012154 CP035042 |  |
|  | CP094828 CP051240 CP048410 CP026747 |  |
|  | CP010869 CP031965 CP029353 |  |
| Lake Baikal | CP048407 CP088968 CP017946 CP017946 | 8 |
|  | CP010978 CP017940 CP048687 CP017946 |  |
| Antarctic lake | CP015583 CP018091 CP015732 CP015583 | 11 |
|  | CP016534 CP075371 CP015731 CP091036 |  |
|  | CP019401 LT629752 CP022957 |  |
| Japan paddy | AP019536 CP078096 CP002727 AP019777 | 16 |
|  | CP049368 CP040128 CP039650 CP009788 |  |
|  | CP049368 LT629758 CP002727 CP002727 |  |
|  | AP019536 CP042466 CP060712 CP022364 |  |
| Northeast paddy | CP063441 CP002446 CP011144 CP010409 | 9 |
|  | CP050114 CP022987 CP050114 CP045238 |  |
|  | CP075052 |  |
| Northeast soil | CP093276 CP022208 CP050520 CP058977 | 12 |
|  | CP050183 CP045423 CP054314 CP101233 |  |
|  | CP077746 CP054254 CP048043 CP048787 |  |
| Glacier | CP012643 CP015150 CP019062 CP021992 | 14 |
|  | CP013236 CP010976 CP062159 CP012643 |  |
|  | CP046105 CP012479 CP003063 AP017423 |  |
|  | CP080597 CP095046 |  |
| Wetland | CP003364 AP024883 CP053538 CP055292 | 13 |
|  | CP014476 CP025001 CP038147 CP003364 |  |
|  | CP053707 CP064653 CP041365 CP041638 |  |
|  | CP090376 |  |

| Habitat | GenBank ID | Number of sequences |
| --- | --- | --- |
| Pacific ocean | CP003179 CP081862 CP002344 CP091912 | 20 |
|  | WP_121520895 CP011011 CP011030 CP002361 |  |
|  | CP003257 CP002344 CP014782 CP031165 |  |
|  | CP029789 CP014796 CP011030 CP085143 |  |
|  | CP022571 CP003257 CP054856 CP091912 |  |
| Atlantic ocean | CP003946 CP002543 CP040367 CP082362 | 13 |
|  | CP072262 CP061846 CP061739 CP059322 |  |
|  | CP000821 CP086344 AP021868 CP060120 |  |
|  | CP031527 |  |
| Arctic ocean | CP010797 CP078076 CP029361 CP041036 | 13 |
|  | CP022434 CP099712 CP012529 CP071868 |  |
|  | CP017965 CP026490 CP072852 CP011494 |  |
|  | CP003735 |  |
| Indian ocean | CP041235 CP091056 CP002683 CP038256 | 15 |
|  | CP041614 CP049990 WP_116038467 CP031598 |  |
|  | CP042862 CP094533 CP012154 CP035042 |  |
|  | CP094828 CP045200 CP031555 |  |
| Lake Baikal | CP048407 CP088968 CP088968 BA000012 | 10 |
|  | CP017946 CP017946 CP054031 CP010978 |  |
|  | CP017940 CP048687 |  |
| Antarctic lake | CP015583 CP015732 CP076547 CP024588 | 13 |
|  | CP016534 CP075371 CP015731 WP_121969742 |  |
|  | WP_016989649 CP077725 CP043650 WP_006989689 |  |
|  | CP016534 |  |
| Japan paddy | AP019536 CP076723 CP002727 CP042466 | 19 |
|  | CP077684 CP054931 AP019777 CP040128 |  |
|  | CP039650 CP044074 CP042466 AP025592 |  |
|  | CP000698 CP054931 CP078096 CP002727 |  |
|  | AP019536 CP022364 WP_075074434 |  |
| Northeast paddy | CP045423 CP063441 CP002446 CP011144 | 9 |
|  | CP010409 CP050114 CP002663 CP050114 |  |
|  | CP045238 |  |
| Northeast soil | CP093276 CP058322 CP088975 AP019632 | 12 |
|  | CP058977 CP019572 CP071696 CP040077 |  |
|  | CP019038 CP002810 CP053289 WP_198304081 |  |
| Glacier | AP022643 CP067394 CP079108 CP012643 | 5 |
|  | CP041401 |  |
| Wetland | CP001338 CP042908 CP003364 CP090029 |  |
|  | CP013297 CP020618 CP081178 CP019162 |  |
|  | CP071060 CP093322 CP063663 CP003364 |  |
|  | CP090376 |  |

**Table S3 Nucleotide sequence fragment information of *phoU* gene in different habitats**

**Table S4 Nucleotide sequence fragment information of *pstS* gene in different habitats**

| Habitat | GenBank ID | Number of sequences |
| --- | --- | --- |
| Pacific ocean | CP003179 CP081862 CP002344 CP011011 | 20 |
|  | CP011030 CP031165 CP029789 CP011030 |  |
|  | CP003257 CP091912 CP000111 CP012299 |  |
|  | CP006269 CP045427 CP072673 CP053084 |  |
|  | CP026530 CP045403 CP024087 CP083954 |  |
| Atlantic ocean | HQ337021 CP003946 CP040367 CP082362 | 13 |
|  | CP072262 CP061846 CP061739 CP059322 |  |
|  | CP086344 CP060120 CP031527 CP050153 |  |
|  | CP053562 |  |
| Arctic ocean | CP001796 CP010797 CP080491 CP090837 | 13 |
|  | CP041036 LR134501 CP099712 CP061739 |  |
|  | CP071868 CP017965 CP026490 CP072852 |  |
|  | CP000447 |  |
| Indian ocean | CP041235 CP091056 CP002683 CP063052 | 15 |
|  | CP038256 CP041614 CP049990 CP097160 |  |
|  | CP069161 CP031598 AP025692 CP042862 |  |
|  | CP007451 CP094533 CP069317 |  |
| Lake Baikal | CP048407 CP061072 CP073761 CP006942 | 12 |
|  | CP060395 CP088968 CP071520 CP052769 |  |
|  | CP017946 CP017946 CP054021 CP081350 |  |
| Antarctic lake | CP015583 CP018091 WP_240519037 CP015732 | 13 |
|  | CP076547 CP095501 CP015583 CP016534 |  |
|  | CP075371 CP015731 CP017774 CP095501 |  |
|  | WP_006989685 |  |
| Japan paddy | AP019536 CP077684 CP002727 CP042466 | 19 |
|  | OW485601 CP039646 CP001124 AP019777 |  |
|  | CP049368 CP040128 CP039650 CP022198 |  |
|  | CP031145 CP009788 AP025592 CP000148 |  |
|  | OW485601 CP040429 CP076723 |  |
| Northeast paddy | CP045423 CP063441 CP011144 CP093331 | 9 |
|  | CP010409 CP014060 CP050114 CP050115 |  |
|  | CP045238 |  |
| Northeast soil | CP093276 CP029378 CP058322 CP088975 | 12 |
|  | CP050520 CP058977 CP019572 CP047176 |  |
|  | CP001052 CP019038 CP002810 CP068985 |  |
| Glacier | CP041401 CP021992 CP017479 CP051685 |  |
|  | CP083954 LT629742 CP053954 CP010945 |  |
|  | CP071868 CP022296 CP019062 CP007595 |  |
|  | CP080597 CP046175 LT799838 | 15 |
| Wetland | CP042908 CP038147 CP090029 CP064653 |  |
|  | CP034683 CP053707 WP_007784642 CP061286 |  |
|  | CP052569 CP089507 AP018439 |  |

| Host source | GenBank ID | Number of sequences |
| --- | --- | --- |
| Bacteria | CP104681 CP050240 CP009781 CP023314 | 25 |
|  | CP002775 LR607330 CP046722 CP026977 |  |
|  | CP047495 CP083737 CP049603 CP027798 |  |
|  | CP011802 CP025738 CP014035 CP048056 |  |
|  | CP011391 AP017991 CP034089 CP002886 |  |
|  | CP083988 CP002869 CP017746 CP029237 |  |
|  | CP069350 |  |
| Uncultured bacteria | WP_055164082 WP_007862287 CP059856 | 20 |
|  | CP102266 BK036781 CP029462 VBB48696 |  |
|  | LR738849 CP036295 CP064353 AM184116 |  |
|  | FO117592 OU461276 CP056809 JX875550 |  |
|  | CP101990 MT002444 CP095412 MN577573 |  |
|  | CP032317 |  |
| Fungi | AP025689 CP011077 CP033149 CP001891 | 12 |
|  | OW969912 CP028212 CP011427 CP054254 |  |
|  | CP013398 CP002013 CP047147 CP095376 |  |
| Phage | NC_047749 NC_047979 JQ067087 HQ317393 | 13 |
|  | FM207411 KF623294 GU071103 NC_048760 |  |
|  | JX483881 KT353109 MK568540 NC_049372 |  |
|  | NC_027989 |  |
| Uncultured phage | MN986607 MN986605 MN986602 MN986589 |  |
|  | MN986578 MN986575 MN986539 MN986518 |  |
|  | MN986520 MN986522 MN986524 MN986526 |  |
|  | MN986592 MN986563 MN986533 MN986524 |  |
|  | MN986527 |  |
| Virus | KM979354 HE600015 EU304328 FN600414 | 17 |
|  | NC_048794 HQ632826 HQ632827 FQ312032 |  |
|  | NC_022343 MN336263 AB231700 OM876856 |  |
|  | NC_048873 EU304328 OM876856 HQ632826 |  |
|  | MK892724 |  |
| Uncultured virus | KX189987 KX189981 KX190026 KX189861 | 30 |
|  | KX189885 KX189863 KX189755 KX189749 |  |
|  | KX189741 KX189672 KX189698 KX189682 |  |
|  | KX189800 KX189859 KX189796 KX189688 |  |
|  | JF964243 JF964234 JF964213 JF964168 |  |
|  | JF964134 JF964108 JF964065 JF964058 |  |
|  | JF964048 JF964035 JF964031 JF964019 |  |
|  | KP784070 KP784048 |  |

**Table S5 Nucleotide sequence fragment information of *phoH* gene from different host sources**

**Table S6 Nucleotide sequence fragment information of *phoU* gene from different host sources**

| Host source | GenBank ID | Number of sequences |
| --- | --- | --- |
| Bacteria | CP034908 CP000084 CP001962 FN557490 | 20 |
|  | FO082843 CP020821 CP020028 CP047019 |  |
|  | LR134349 CP045380 CP040500 CP001959 |  |
|  | CP025738 CP014035 AP022868 CP002886 |  |
|  | LT629687 CP029237 AP025014 CP001154 |  |
| Uncultured bacteria | CP003920 AP017459 EU519202 KF124155 | 22 |
|  | CP049781 HF545616 CP102938 EEG47472 |  |
|  | CP041666 AP018532 CP102279 LT635479 |  |
|  | LR813084 OU461276 CAG1064781 CP036259 |  |
|  | AP018794 CP010802 CP045144 CP052058 |  |
|  | AP018532 CP085928 |  |
| Fungi | CP010026 CP053027 CP013404 CP033631 | 14 |
|  | CP044409 CP013236 CP095376 AP025944 |  |
|  | CP086201 CP049978 AP014690 CP076444 |  |
|  | CP015250 CP082346 |  |
| Archaea | CP001956 CP001365 CP003243 CP003412 | 24 |
|  | CP001932 CP002062 AE000782 LR698974 |  |
|  | CP081183 CP019327 CP003050 CP064788 |  |
|  | CP001398 HF571520 CP104300 CP039139 |  |
|  | CP023154 CP033241 LR216287 CP002838 |  |
|  | CP003321 AP012489 CP062310 CP007493 |  |
| Uncultured archaea | FP565147 KF900842 CR937011 CP076019 | 12 |
|  | VVB96190 VVB56369 VVB71528 CP034267 |  |
|  | CP009479 EU016611 CP058335 VVB85501 |  |

**Table S7 Nucleotide sequence fragment information of *pstS* gene from different host sources**

| Host source | GenBank ID | Number of sequences |
| --- | --- | --- |
| Bacteria | CP104788 CP049140 CP034908 AP009493 | 21 |
|  | CP100764 CP027107 BX251411 LR134369 |  |
|  | CP065838 CP026977 CP043318 CP003244 |  |
|  | CP011802 CP025738 CP014035 AP017991 |  |
|  | CP049767 CP002886 AP022871 CP001154 |  |
|  | EF601159 |  |
| Uncultured bacteria | MN433467 GU055829 GU055791 AP025568 | 25 |
|  | CP102267 AP018536 FP929054 LR699012 |  |
|  | AP018532 CP094466 LT990039 CP094466 |  |
|  | CP029462 CP017420 OU461276 CP077684 |  |
|  | SCM81920 SBV92827 CP048436 WP_262538581 |  |
|  | VBB43965 SLM17497 CP085135 CP016952 |  |
|  | JQ844215 |  |
| Fungi | XM_016754687 CP010026 CP102482 CP010026 | 14 |
|  | CP050958 CP046047 CP095376 CP018846 |  |
|  | CP025034 CP006704 AP014690 CP014505 |  |
|  | CP024655 CP045216 |  |
| Phage | HQ634176 GU071097 LR798288 HQ632825 | 11 |
|  | HQ337021 GU071098 GU071099 GU071106 |  |
|  | HQ632825 GU071094 GU071095 |  |
| Archaea | CP000968 CP033241 CP001401 CP000504 | 27 |
|  | BA000002 CP010868 CP009961 CP003531 |  |
|  | CP002838 CP003098 CP030846 CP002363 |  |
|  | CP001398 LT549890 HF571520 CP104300 |  |
|  | CP078063 CP023154 LT549890 LR216287 |  |
|  | CP002838 CP003321 CP003423 AP012489 |  |
|  | CP062310 GBE54858 CP007493 |  |

**Table S8 Amino acid sequence fragment information of *phoH* gene in different habitats**

| Habitat | GenBank ID | Number of sequences |
| --- | --- | --- |
| Ocean | WP_043744270 KNG94448 WP_057790249 | 11 |
|  | QEW25897 RCK53892 WP_007088434 |  |
|  | WP_045996657 KFI23793 AEH32273 |  |
|  | YP_008130050 YP_007673009 |  |
| Freshwater lake | WP_116899892 PHY05758 MSU27622 | 20 |
|  | WP_060697380 WP_105019920 TWX40560 |  |
|  | QDB71460 WP_002739950 WP_069475387 |  |
|  | GDX75700 GDX37826 RLT18466 |  |
|  | WP_026786245 MBJ7344042 TBW36478 |  |
|  | KJR43636 KJJ84727 WP_077510950 |  |
|  | WP_077354667 WP_041114740 |  |
| Paddy | WP_136704808 NBD01285 NJQ19717 | 12 |
|  | ANW11874 ANW11639 ANW11623 |  |
|  | ANW11498 WP_226239478 WP_040042455 |  |
|  | WP_099820014 WP_000840516 GIE82658 |  |
| Glacier | KFF60682 WP_091197128 WP_143571183 | 10 |
|  | WP_149098671 WP_135498675 WP_192184348 |  |
|  | WP_088457985 WP_054509179 WP_143373371 |  |
|  | PPL18695 |  |
| Wetland | WP_059119829 WP_135528831 WP_125422631 | 17 |
|  | WP_002449734 WP_145306896 AYM46577 |  |
|  | AYM46593 AYM46626 AYM46636 |  |
|  | AYM46646 AYM46656 AYM46661 |  |
|  | AYM46666 AYM46669 AYM46679 |  |
|  | AYM46675 AYM46677 |  |

**Table S9 Amino acid sequence fragment information of *phoU* gene in different habitats**

| Habitat | GenBank ID | Number of sequences |
| --- | --- | --- |
| Ocean | WP_130990937 WP_223039651 WP_009824650 | 20 |
|  | WP_119397051 WP_126409254 WP_138252055 |  |
|  | WP_025911582 WP_039101073 KAA1299224 |  |
|  | WP_148120654 WP_014872892 WP_007586465 |  |
|  | WP_041412865 WP_111605856 WP_006125649 |  |
|  | WP_008888333 WP_067027880 WP_145036288 |  |
|  | WP_019274141 WP_004587621 |  |
| Freshwater lake | WP_116900279 MSO51429 MSQ86129 | 16 |
|  | MSU28359 MSU54027 GDY19105 |  |
|  | GDX99794 GDX79013 RLT56974 |  |
|  | RLT49249 RLT19484 WP_002737378 |  |
|  | WP_009096049 QSV65073 WP_131307714 |  |
|  | RNJ62881 |  |
| Paddy | WP_223909086 WP_217288204 AKB62529 | 15 |
|  | WP_176062686 WP_212785789 WP_093619603 |  |
|  | WP_164202239 WP_149307031 WP_028367730 |  |
|  | WP_125292906 WP_010965016 WP_136705534 |  |
|  | WP_109496892 WP_106337619 AEW75964 |  |
| Glacier | WP_143572051 WP_088457058 WP_108439046 | 15 |
|  | WP_149097673 WP_106760022 GBU08554 |  |
|  | WP_126744081 WP_107939313 WP_215342250 |  |
|  | WP_133107968 WP_130102417 WP_120165622 |  |
|  | WP_091198495 WP_128604582 WP_007896483 |  |
| Wetland | WP_014154896 WP_196434235 WP_126790107 | 15 |
|  | RPJ62498 RPJ60804 RPJ51892 |  |
|  | RPJ34572 MBS1219722 WP_008150055 |  |
|  | WP_002455359 WP_018267050 WP_071397746 |  |
|  | WP_037001617 WP_088248791 WP_005334097 |  |

**Table S10 Amino acid sequence fragment information of *pstS* gene in different habitats**

| Habitat | GenBank ID | Number of sequences |
| --- | --- | --- |
| Ocean | WP_100883503 WP_142873299 WP_130050724 | 16 |
|  | WP_126409257 TET66969 PCH84862 |  |
|  | WP_013857520 WP_135795249 WP_226066238 |  |
|  | WP_007102238 WP_186508784 WP_121134176 |  |
|  | WP_111605853 WP_101086502 WP_144041856 |  |
|  | NEE39157 |  |
| Freshwater lake | PHX88239 WP_070199699 WP_034778123 | 15 |
|  | ONI49819 WP_002778969 WP_185238352 |  |
|  | WP_002736281 GBL44175 GDX85335 |  |
|  | QSV61942 RLT36856 WP_106309370 |  |
|  | WP_215298631 WP_215320468 MCA2723226 |  |
| Paddy | WP_223906793 WP_190601887 WP_152588357 | 15 |
|  | WP_239143192 WP_136704841 WP_106337623 |  |
|  | WP_231908216 WP_152587995 WP_221048257 |  |
|  | WP_151156361 WP_150041494 THF53945 |  |
|  | WP_217288203 WP_212786623 WP_028367726 |  |
| Glacier | WP_143572047 WP_134574588 WP_134362254 | 15 |
|  | WP_104923883 WP_106757430 WP_000871817 |  |
|  | WP_126744078 WP_131062509 WP_129473901 |  |
|  | WP_108634289 GBU12545 WP_108439049 |  |
|  | WP_149097677 GBU15517 WP_099914477 |  |
| Wetland | WP_079439875 WP_223923598 RPI84074 | 15 |
|  | RPI62292 RPI25102 RPI16111 |  |
|  | RPI08439 WP_010465320 RPH91166 |  |
|  | WP_127684741 WP_196435401 RPH94475 |  |
|  | HBU0830504 WP_215390759 WP_012319312 |  |

**Table S11 Amino acid sequence fragment information of *phoH* gene from different host source**

| Host source | GenBank ID | Number of sequences |
| --- | --- | --- |
| Bacteria | AHW58931 WP_045026729 WP_038555548 | 20 |
|  | WP_073166322 WP_107823903 OFX83821 |  |
|  | KPJ86234 MBL7933316 OFY82246 |  |
|  | OFZ12809 MBN9292730 WP_003228522 |  |
|  | WP_010789239 AFH69812 WP_013026125 |  |
|  | WP_004147894 AJE55333 WP_038661516 |  |
|  | WP_014515835 WP_054538067 |  |
| Uncultured bacteria | VBB48696 BAL56418 QIM10836 | 20 |
|  | BAL56100 AAS07905 QGT51276 |  |
|  | AMK59169 WP_004281434 WP_075703530 |  |
|  | WP_118064259 WP_022118631 WP_216412270 |  |
|  | WP_026367772 CBX29940 ADI20259 |  |
|  | WP_075756149 WP_046671327 SBV35900 |  |
|  | QJR98039 AIF26842 |  |
| Fungi | WP_155011535 WP_060856957 AGI90450 | 10 |
|  | RZD82391 WP_028196368 WP_021508569 |  |
|  | WP_021509913 WP_028196368 WP_094111648 |  |
|  | WP_079257598 |  |
| Phage | YP_009010160 YP_008125829 YP_009283581 | 20 |
|  | YP_003097238 YP_009302685 YP_008771079 |  |
|  | YP_008130050 YP_007673009 YP_009822813 |  |
|  | YP_007518057 YP_009206870 YP_009838400 |  |
|  | BBA65336 YP_006908240 YP_009321122 |  |
|  | YP_008431127 YP_008431127 AOV62448 |  |
|  | YP_009324389 YP_009006403 |  |
| Uncultured phage | QPF70332 QPF70331 QPF70420 | 11 |
|  | QPF70416 QPF70408 QPF70383 |  |
|  | QPF70386 QPF70353 QPF70415 |  |
|  | QPF70345 QPF70347 |  |
| Virus | YP_007676098 YP_009168480 YP_004062114 | 3 |
| Uncultured virus | ANW11840 AKV56932 ANW11849 | 26 |
|  | ANW11870 ANW11849 ANW11855 |  |
|  | ANW11680 ANW11669 ANW11639 |  |
|  | ANW11701 ANW11655 ANW11515 |  |
|  | ANW11616 ANW11505 ANW11553 |  |
|  | ANW11596 ANW11498 ANW11500 |  |
|  | ANW11528 ANW11525 ANW11564 |  |
|  | AEQ27568 AEQ27560 AEQ27539 |  |
|  | AEQ27545 AEQ27550 |  |

**Table S12 Amino acid sequence fragment information of *phoU* gene from different host**

**sources**

| Host source | GenBank ID | Number of sequences |
| --- | --- | --- |
| Bacteria | WP_015359861 WP_003229617 WP_024079144 | 15 |
|  | EEJ59256 WP_012101741 AAO43117 |  |
|  | WP_014335892 WP_012696370 WP_000946463 |  |
|  | WP_013023944 WP_002747618 WP_002965206 |  |
|  | WP_217220638 WP_014653301 WP_007053841 |  |
| Uncultured bacteria | EKD88295 WP_006570822 WP_042396988 | 15 |
|  | KXA62395 VBB42785 SUS06994 |  |
|  | WP_075752637 WP_100080842 SBW08648 |  |
|  | SBS72260 WP_007209708 EEG47472 |  |
|  | WP_173736609 SCJ87914 MBD9090365 |  |
| Fungi | PRZ45675 WP_006682666 WP_159508614 | 9 |
|  | WP_028195888 WP_003949791 WP_145485837 |  |
|  | WP_028199154 WP_028197469 WP_025496623 |  |
| Archaea | AAG18994 WP_011012147 WP_058984700 | 15 |
|  | WP_014587662 WP_058994896 WP_058571735 |  |
|  | WP_049900089 WP_049894332 WP_048173074 |  |
|  | WP_013683552 WP_011324430 WP_008417759 |  |
|  | WP_048091270 WP_023394216 WP_007260123 |  |
| Uncultured archaea | AIF02137 AIF00448 AIF06008 | 7 |
|  | AIE96109 CBH38055 CBH38056 |  |
|  | AIF16166 |  |

**Table S13 Amino acid sequence fragment information of *pstS* gene from different host sources**

| Host source | GenBank ID | Number of sequences |
| --- | --- | --- |
| Bacteria | WP_014335888 WP_096925454 WP_012696009 | 15 |
|  | WP_004145003 WP_151290421 WP_052369453 |  |
|  | WP_215539205 WP_013023940 HBO2405901 |  |
|  | WP_012907983 ADO95180 ABU55413 |  |
|  | WP_072166779 WP_014477353 WP_012101737 |  |
| Uncultured bacteria | VUZ23184 CAG8999406 SPD74619 | 15 |
|  | SCM81920 SBV92827 WP_238722861 |  |
|  | SCI20194 MBS6878491 SCJ87862 |  |
|  | SCJ80296 MBP8841945 WP_154044993 |  |
|  | SCJ21688 WP_054327438 WP_021748867 |  |
| Fungi | WP_033759311 WP_046565172 WP_060818870 | 7 |
|  | WP_028199158 WP_025496808 WP_021506928 |  |
|  | WP_104085891 |  |
| Phage | YP_008130020 YP_214479 YP_004324340 | 5 |
|  | YP_214479 YP_214730 |  |
| Archaea | WP_112149348 WP_192819317 WP_166285185 | 15 |
|  | BAA78954 WP_081227387 SMH70285 |  |
|  | WP_012309464 WP_014026439 TMI72203 |  |
|  | TMA02366 TDA29831 WP_247140516 |  |
|  | WP_121561594 ACP48301 WP_202320032 |  |
